# TRANSCRIPTOME ALTERATIONS ASSOCIATED WITH AGE-RELATED DECLINE IN PHYSICAL FUNCTION.

**DOI:** 10.1093/geroni/igz038.3199

**Published:** 2019-11-08

**Authors:** Ted G Graber, Rosario Marota, Jill Thompson, Steve Widen, Blake Rasmussen

**Affiliations:** 1 East Carolina University, Department of Physical Therapy, Greenville, North Carolina, United States; 2 University of Texas Medical Branch, Department of Nutrition and Metabolism, Galveston, Texas, United States

## Abstract

One inevitable consequence of the effect of age on our bodies is the graduated deterioration of physical function and exercise capacity, driven, in part by the adverse effect of age on muscle tissue. Our primary purpose was to determine the relationship between patterns of gene expression in skeletal muscle and loss of physical function. We hypothesized that some genes that change expression with age would correlate with functional decline, or conversely with preservation of function. Male C57Bl/6 mice [adults (6-7 months old, n=9), older (24-25 months old, n=9), and elderly (28+ months of age, n=9) were tested for physical ability using a comprehensive functional assessment battery [CFAB, a composite scoring system: comprised of the rotarod (overall motor function), grip strength (fore-limb strength), inverted cling (4-limb strength/endurance), voluntary wheel running (activity rate/volitional exercise), and treadmill tests (endurance)]. We extracted RNA from the tibialis anterior muscles, ran RNAseq to examine the transcriptome using an Illumina NextSeq 550, comparing adults (n=7) to older (n=7) and elderly mice (n=9). Age resulted in gene expression differences of 1.5 log2 fold change or greater (p<0.01) in 46 genes in the older mice and in 252 genes in the elderly (both compared to adults). Current ongoing work is examining the physiological relevance of these genes to age-related loss of physical function. We are in the process of using linear regression to determine which of the genes with age-related changes in expression are associated (R>0.5 and p<0.05) with functional status as measured by CFAB.

